# Chemical Science moves to weekly issues!

**DOI:** 10.1039/c7sc90079a

**Published:** 2017-12-15

## Abstract

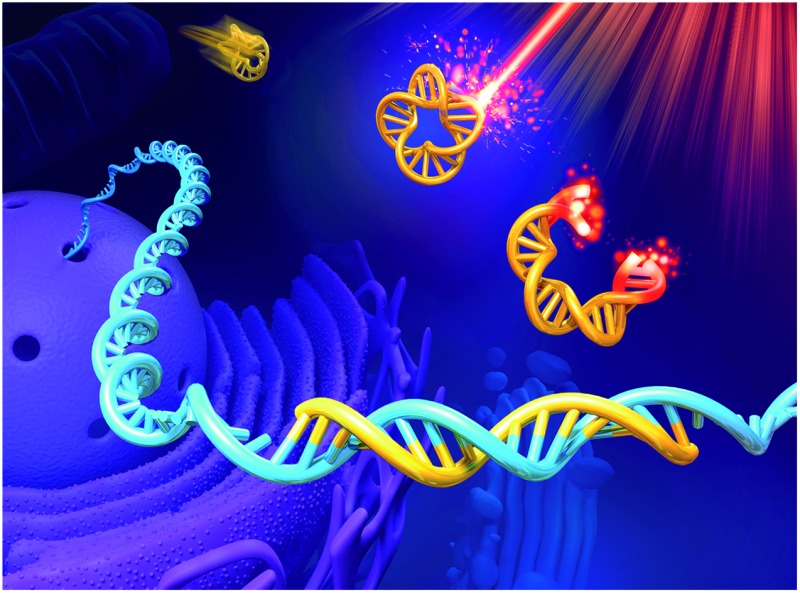
2018 starts with an exciting new development for *Chemical Science*, with a move to weekly publication.

## 

2018 promises to be another exciting year for *Chemical Science*, with one of the biggest changes to the journal being a move to publishing weekly issues.

## Why are we doing this?

The main reason is to help us keep up with the increasing number of articles published in the journal. Since 2010, we have been growing steadily and published nearly 1000 articles in 2017. This means the journal has just simply outgrown twelve monthly issues, and in the interests of our authors and readers, we want to provide our articles in more bite-size and accessible chunks.

A key benefit for authors of weekly publication will be the more rapid assignment of page numbers to their articles. A shorter table of contents alert, sent weekly, will help researchers discover the latest cutting-edge developments more quickly, and readers will also have shorter contents lists to browse. Authors will also find that their articles appear sooner in Web of Science, Scopus and PubMedCentral.

*Chemical Science* is the home for only the most cutting-edge solutions to today’s global challenges. This increase in the number of issues doesn’t necessarily indicate that we are planning a future increase in the number of articles. We will still be looking to publish only findings of the most exceptional significance from across the chemical sciences. However, if we continue to receive more articles that meet this very high standard, then the journal will be able to develop further in a weekly format, without delaying our authors.

## Leading the way in open access

In January 2015, *Chemical Science* became the world’s first high quality gold open access chemistry journal.

We are delighted to say it is free to read, and free to publish in *Chemical Science*, as we continue to waive the article-processing charge for our authors. At the Royal Society of Chemistry we aim to connect the world with the chemical sciences, and one of the ways we can do this is through making our flagship journal accessible to all.

We are also committed to increasing the visibility of our articles, and so the journal is now fully indexed in PubMedCentral and also listed in the Directory of Open Access Journals (DOAJ).

## What has everyone been reading in 2017?

The table below reveals a selection of our most-read articles published in 2017. Have you seen them yet? Congratulations to all the authors for this excellent achievement.

**Table 1:** A selection of our most-read articles in 2017

Title	Authors	DOI
Antibody fragments as nanoparticle targeting ligands: a step in the right direction	Daniel A. Richards*, Antoine Maruani and Vijay Chudasama*	DOI: 10.1039/c6sc02403c (Perspective), *Chem. Sci*., 2017, **8**, 63–77
DNA-barcoded labeling probes for highly multiplexed exchange-PAINT imaging	Sarit S. Agasti, Yu Wang, Florian Schueder, Aishwarya Sukumar, Ralf Jungmann* and Peng Yin*	DOI: 10.1039/c6sc05420j (Edge Article) *Chem. Sci*., 2017, **8**, 3080–3091
Single-atom catalysts for CO_2_ electroreduction with significant activity and selectivity improvements	Seoin Back, Juhyung Lim, Na-Young Kim, Yong-Hyun Kim and Yousung Jung*	DOI: 10.1039/c6sc03911a (Edge Article) *Chem. Sci.*, 2017, **8**, 1090–1096
Metal-free direct alkylation of unfunctionalized allylic/benzylic sp^3^ C–H bonds *via* photoredox induced radical cation deprotonation	Rong Zhou, Haiwang Liu, Hairong Tao, Xingjian Yu and Jie Wu*	DOI: 10.1039/c7sc00953d (Edge Article) *Chem. Sci.*, 2017, **8**, 4654–4659
Sub-1.1 nm ultrathin porous CoP nanosheets with dominant reactive {200} facets: a high mass activity and efficient electrocatalyst for the hydrogen evolution reaction	Chao Zhang, Yi Huang, Yifu Yu, Jingfang Zhang, Sifei Zhuo and Bin Zhang*	DOI: 10.1039/c6sc05687c (Edge Article) *Chem. Sci.*, 2017, **8**, 2769–2775
Recent developments in and perspectives on three-coordinate boron materials: a bright future	Lei Ji, Stefanie Griesbeck and Todd B. Marder*	DOI: 10.1039/c6sc04245g (Perspective) *Chem. Sci.*, 2017, **8**, 846–863
Fully conjugated ladder polymers	Jongbok Lee, Alexander J. Kalin, Tianyu Yuan, Mohammed Al-Hashimi and Lei Fang*	DOI: 10.1039/c7sc00154a (Perspective) *Chem. Sci.*, 2017, **8**, 2503–2521
Copper(i)-catalyzed sulfonylative Suzuki–Miyaura cross-coupling	Yiding Chen and Michael C. Willis*	DOI: 10.1039/c6sc05483h (Edge Article) *Chem. Sci.*, 2017, **8**, 3249–3253
ANI-1: an extensible neural network potential with DFT accuracy at force field computational cost	J. S. Smith, O. Isayev* and A. E. Roitberg	DOI: 10.1039/c6sc05720a (Edge Article) *Chem. Sci.*, 2017, **8**, 3192–3203
Thermally activated delayed fluorescent phenothiazine–dibenzo[*a*,*j*]phenazine–phenothiazine triads exhibiting tricolor-changing mechanochromic luminescence	Masato Okazaki, Youhei Takeda, Przemyslaw Data*, Piotr Pander, Heather Higginbotham, Andrew P. Monkman and Satoshi Minakata	DOI: 10.1039/c6sc04863c (Edge Article) *Chem. Sci.*, 2017, **8**, 2677–2686
A unified photoredox-catalysis strategy for C(sp^3^)–H hydroxylation and amidation using hypervalent iodine	Guo-Xing Li, Cristian A. Morales-Rivera, Fang Gao, Yaxin Wang, Gang He, Peng Liu* and Gong Chen	DOI: 10.1039/c7sc02773g (Edge Article) *Chem. Sci.*, 2017, **8**, 7180–7185
Double quick, double click reversible peptide “stapling”	Claire M. Grison, George M. Burslem, Jennifer A. Miles, Ludwig K. A. Pilsl, David J. Yeo, Zeynab Imani, Stuart L. Warriner, Michael E. Webb and Andrew J. Wilson*	DOI: 10.1039/c7sc01342f (Edge Article) *Chem. Sci.*, 2017, **8**, 5166–5171
Room temperature decarboxylative cyanation of carboxylic acids using photoredox catalysis and cyanobenziodoxolones: a divergent mechanism compared to alkynylation	Franck Le Vaillant, Matthew D. Wodrich and Jérôme Waser*	DOI: 10.1039/c6sc04907a (Edge Article) *Chem. Sci.*, 2017, **8**, 1790–1800
Mild, visible light-mediated decarboxylation of aryl carboxylic acids to access aryl radicals	L. Candish, M. Freitag, T. Gensch and F. Glorius*	DOI: 10.1039/c6sc05533h (Edge Article) *Chem. Sci.*, 2017, **8**, 3618–3622
Dual cobalt–copper light-driven catalytic reduction of aldehydes and aromatic ketones in aqueous media	Arnau Call, Carla Casadevall, Ferran Acuña-Parés, Alicia Casitas and Julio Lloret-Fillol*	DOI: 10.1039/c7sc01276d (Edge Article) *Chem. Sci.*, 2017, **8**, 4739–4749

## Welcome to our new Associate Editors

We are delighted to welcome Professor Mircea Dincă and Professor Vincent Artero as new Associate Editors for the journal. Please do consider sending them your next paper.

Mircea Dincă is Professor in the Department of Chemistry at MIT and his research interests lie in the synthesis of new multifunctional materials for applications in electrical and electronic devices, heterogeneous catalysis, and various uses in clean and renewable energy. In recognition of Mircea’s group’s research, he has been awarded the Alan T. Waterman Award from the NSF in 2016 and the ACS Award in Pure Chemistry in 2018, among several others. Mircea is keen to receive submissions in his area of expertise, particularly MOF-related and multi-functional material research.
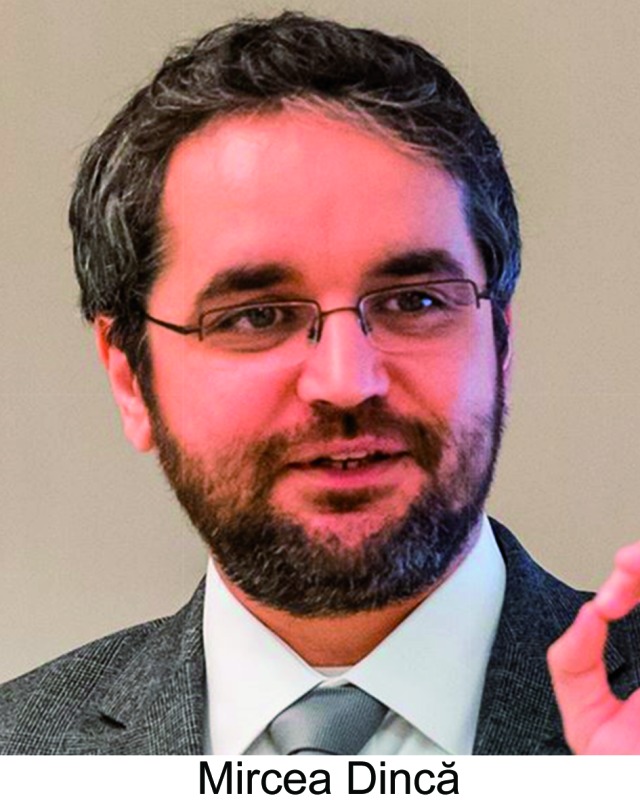


Vincent Artero is Research Director in the Laboratory of Chemistry and Biology of Metals (a research unit cooperated by CEA, CNRS and Univ. Grenoble Alpes) in Grenoble, France. His research interests are in bioinspired chemistry and artificial photosynthesis. His group targets the structural and functional modelisation of hydrogenases, the design of artificial organometallic proteins and the design of novel nanomaterials for hydrogen photo- and electro-production, hydrogen oxidation and CO_2_ reduction. Vincent Artero received the “Grand Prix Mergier-Bourdeix de l’Académie des Sciences” in 2011. Vincent is keen to receive submissions in his area of expertise, particularly in bioinspired chemistry, artificial photosynthesis and renewable energy sources.
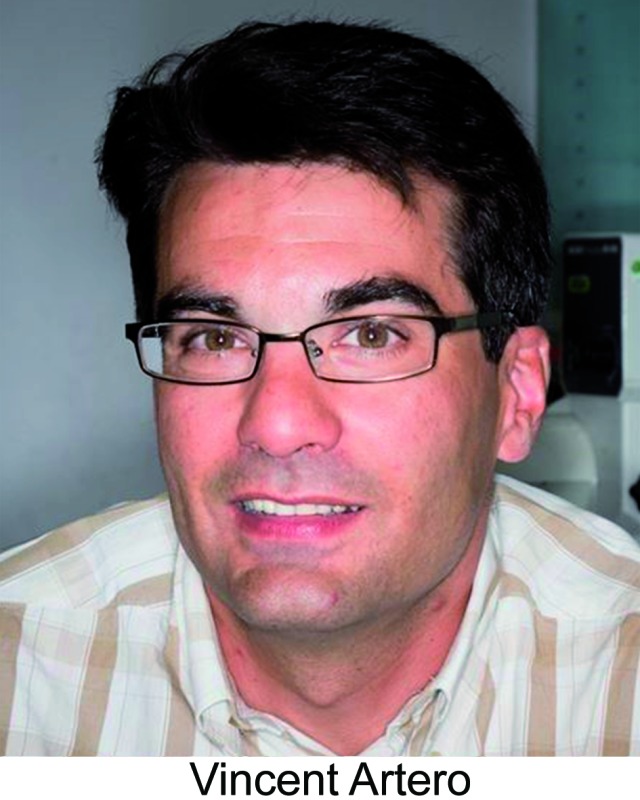


## Thank you to our boards, authors and reviewers

We are striving to improve *Chemical Science’s* quality, impact and service, and we can only do this with the support of our Editorial and Advisory Boards, authors and reviewers. You are critical for the journal’s success and I’d like to sincerely thank you for this.

We recognised our outstanding reviewers of 2016 as chosen by the Editorial team, by publishing a list of them in the journal in the spring of 2017.^1^ We’ll be doing this again this year, so watch out for our outstanding reviewers of 2017 coming soon.

We hope to meet many of you in the year ahead and, as always, we would be pleased to receive your feedback and suggestions.

On behalf of the *Chemical Science* Editorial team, a very happy New Year!

May Copsey

Executive Editor, *Chemical Science*.
